# Rapid Detection Methods for Asphalt Pavement Thicknesses and Defects by a Vehicle-Mounted Ground Penetrating Radar (GPR) System

**DOI:** 10.3390/s16122067

**Published:** 2016-12-06

**Authors:** Zehua Dong, Shengbo Ye, Yunze Gao, Guangyou Fang, Xiaojuan Zhang, Zhongjun Xue, Tao Zhang

**Affiliations:** 1 Institute of Electronics, Chinese Academy of Science, Beijing 100190, China; shengboye@163.com (S.Y.); gyze@163.com (Y.G.); gyfang@mail.ie.ac.cn (G.F.); xjzhang@mail.ie.ac.cn (X.Z.); 2 University of Chinese Academy of Science, Beijing 100149, China; 3 Beijing Road Engineering Quality Supervision Station, Beijing 100076, China; xuezhongjun@126.com (Z.X.); gonglujiance@126.com (T.Z.)

**Keywords:** vehicle-mounted GPR system, asphalt layer thickness, top surface asphalt layer, layer localization method, degree of compaction, delamination at interfaces

## Abstract

The thickness estimation of the top surface layer and surface layer, as well as the detection of road defects, are of great importance to the quality conditions of asphalt pavement. Although ground penetrating radar (GPR) methods have been widely used in non-destructive detection of pavements, the thickness estimation of the thin top surface layer is still a difficult problem due to the limitations of GPR resolution and the similar permittivity of asphalt sub-layers. Besides, the detection of some road defects, including inadequate compaction and delamination at interfaces, require further practical study. In this paper, a newly-developed vehicle-mounted GPR detection system is introduced. We used a horizontal high-pass filter and a modified layer localization method to extract the underground layers. Besides, according to lab experiments and simulation analysis, we proposed theoretical methods for detecting the degree of compaction and delamination at the interface, respectively. Moreover, a field test was carried out and the estimated results showed a satisfactory accuracy of the system and methods.

## 1. Introduction

The surface layer of asphalt pavement is a multilayer structure. It commonly has three sub-layers: the top surface layer, the intermediate surface layer and the following surface layer. The thicknesses of the surface layer and the top surface layer have great importance on quality condition and maintenance of asphalt pavements [1]. In addition, road defects, such as inadequate compaction and delamination at the interface, also have significant impact on the quality and life of asphalt pavements. Therefore, it is of great importance to detect the thicknesses and defects of asphalt pavements rapidly and accurately [2].

Conventionally, the thickness and defect of asphalt pavement in the field are assessed by direct destructive methods, including digging test-pits or by extracting cores. However, these kinds of methods have inherent drawbacks, such as being intrusive, time-consuming and discontinuous [3]. To overcome these limitations, non-destructive techniques applications are widely used. The ground penetrating radar (GPR) technique, as an example of a non-destructive technique, has been applied for pavement investigations in recent years due to its advantages of high speed data collection and a continuous profile [4,5]. The GPR technique can estimate the thickness of asphalt pavement and identify some underground distresses using the propagation and reflection of electromagnetic waves in underground medium [6]. With respect to the convenience and efficiency of GPR application, lots of vehicle-mounted GPR systems were developed and applied [7,8,9,10]. Allowing for the specific requirements in pavement detection, some factors should be considered for the GPR system: extensive frequencies for both deep and shallow structures; effective and accurate localization; clear in-place surface photos.

Currently, most studies of the thickness estimation by GPR are concentrated on the thickness of asphalt surface layers. Al-Qadi and Lahouar used the GPR reflected signal and different data processing techniques to estimate the dielectric properties of pavement structures, and then determined their thicknesses [11]. Additionally, Liu and Sato presented a ground penetrating radar (GPR) system, which used an antenna array for in-situ measurements of the thickness and dielectric permittivity of an asphalt pavement layer [12]. Besides, Hu et al. also determined the pavement thickness using a ground-coupled GPR [13]. Nevertheless, the thickness estimation of the thin top surface layer was still a difficult problem due to the limitation of GPR resolution and little permittivity difference between two asphalt sub-layers [14]. The performances of five common algorithms were compared in terms of resolution power as well as root-mean-square error on the estimated thickness of thin-pavement [15]. Li used independent component analysis (ICA) and a pattern search method to separate thin surface asphalt layers, but the rate of operation of the multi-iteration was too low [16]. In addition, the overlapped interface was identified in the signal by applying regularized deconvolution and the L-curve method [17]. However, all of the above methods studied only one signal trace and failed to consider and show the performance of the whole survey-line in the GPR profile.

Furthermore, many previous studies examined the parameters and quality of asphalt pavement, such as air voids and moisture [18,19,20,21,22,23,24]. However, two types of asphalt pavement distresses, i.e., inadequate compaction and delamination at interface, were studied in this paper. The most similar studies to compaction degree are density and air voids estimation. Al-Qadi et al. used GPR to measure in-situ asphalt mixture densities on the basis of three prediction models [25]. The accuracy of density prediction was also evaluated to be better than with the nuclear gauge [26]. Besides, Kassem et al. proved that there was very good correlation between the density and permittivity obtained from GPR [27]. Similarly, Plati et al. argued that the GPR coupled with novel algorithms could be an effective tool to improve the asphalt mix compaction process and assessment of in situ density [28]. However, all of these studies were carried out on the basis of density prediction models that required some prior typical parameters. Furthermore, the delamination at interface was also hard to detect because the air gaps are usually less than 1 cm, which would not cause great changes in GPR profile. Consequently, little research has been conducted with respect to the detection of delamination at interface by GPR method.

In this paper, a newly-developed vehicle-mounted GPR detection system was introduced. The system could provide the accurate location and surface photos for a certain GPR signal. We extracted the thin top surface layer from GPR data that had an insufficient resolution on the basis of a horizontal high-pass filter and a modified layer localization method, and then the thickness of surface layer and top surface layer could be obtained. Besides, we developed detection methods for possible poorly compacted area and delamination at interface. Finally, a field test was carried out and the estimated results showed a satisfactory accuracy, which proved that the system and methods could be applied for the detection of thicknesses and defects of asphalt pavement.

## 2. Instruments and Methods

### 2.1. GPR Systems

**Figure 1:**
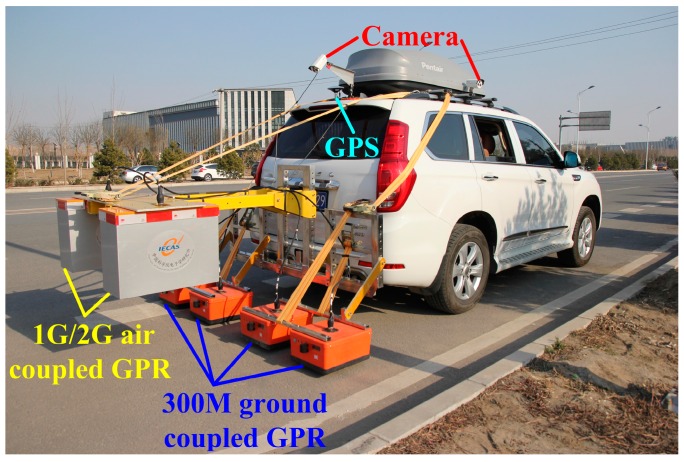
The appearance of the vehicle-mounted GPR detection system.

A novel vehicle-mounted GPR detection system was developed to detect the thicknesses and quality condition of asphalt pavement. The GPR system consists of two air-coupled GPRs, four 300 M ground-coupled GPRs, a GPS and two cameras, as shown in Figure 1. The center frequencies of the left air-coupled GPR, the right air-coupled GPR and the four ground-coupled GPRs are 1 GHz, 2 GHz and 300 MHz, respectively.

One G and two G air-coupled GPRs were mounted on the back of the vehicle (50 cm above the ground), which aimed to detect the defects in the surface asphalt layer and estimate the thicknesses of both the surface layer and top surface layer. Four 300 M ground-coupled GPRs were used for detecting the defects in the base course and sub-base course. GPS was used for localizing every trace of the GPR data. Two cameras on the back and on the right side could collect pictures of the surface conditions and stake numbers, respectively, which were useful for the observation and monitoring of pavement quality condition.

All the onboard instruments, including GPRs, GPS and cameras, could work simultaneously during the detection and all the data were synchronized according to the location and time. As a result, the comprehensive detection for the quality condition of asphalt pavement could be realized using this vehicle-mounted system with a maximum detection speed up to 80 km/h. One trace per 10 cm could be collected when the vehicle came up to the maximum detection speed. In this paper, for the surface asphalt layer detection purpose, only the data of the two G air-coupled GPR, GPS and cameras were employed.

### 2.2. Signal Processing Methods

Generally, asphalt pavements distributed in the city are exposed to certain amounts of electromagnetic interference. The received signals have low signal-to-noise ratio (SNR) because of the radar system noise and out-of-band environment noise. Thus, it was essential to improve the quality of the radar signals that adopting a series of processing methods before the evaluation of pavement thickness and condition. The direct-current (DC) removing, system calibration, filtering and amplitude compensation were employed to suppress the radar system noise, as usually used for general GPR applications. We also used a horizontal high pass filter to extract the thin top surface layer from the GPR data.

#### 2.2.1. Direct-Current (DC) Removal

Direct-current (DC) components came from the oscillation at low frequency during the saturation recovery procedure of the receiver [29]. They should be removed before subsequent processing because they don’t indicate any useful information. The DC removal method was to slide a window from beginning to end of one trace, and then compute the average within the window and subtract the average from the center value of the window, which could be expressed as Equation (1):

(1) $$Y_{\left(n\right)}=y_{c}-\frac{1}{N}\sum_{1}^{N}y(n)$$

where, $y_{c}$ and $Y_{\left(n\right)}$ represent the value before and after processing. y(n) represents the raw LPR data, and *N* is the window length.

#### 2.2.2. System Calibration

**Figure 2:**
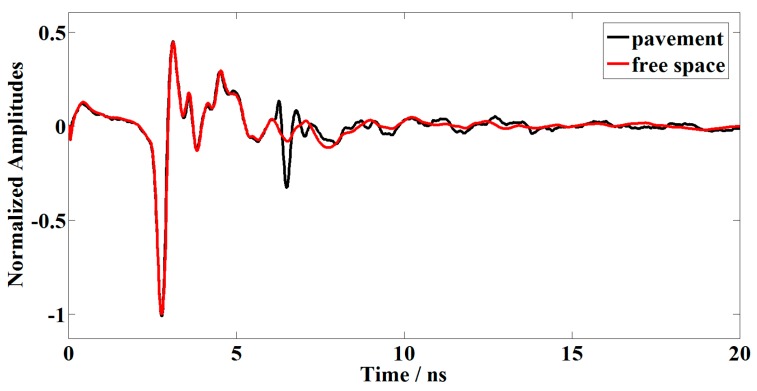
The calibration signal that collected in the air without target (**the red line**) and the GPR data that collected on the pavement (**the black line**).

In order to suppress the noise generated from GPR system, we designed a GPR system calibration experiment which collected calibration signals in the air without target (Figure 2). The calibration signals could be regarded as the aggregation of direct-wave and system noise. Therefore, we subtracted the calibration signal from GPR data that collected on the asphalt pavement and obtained the processed signals.

#### 2.2.3. Filtering

**Figure 3:**
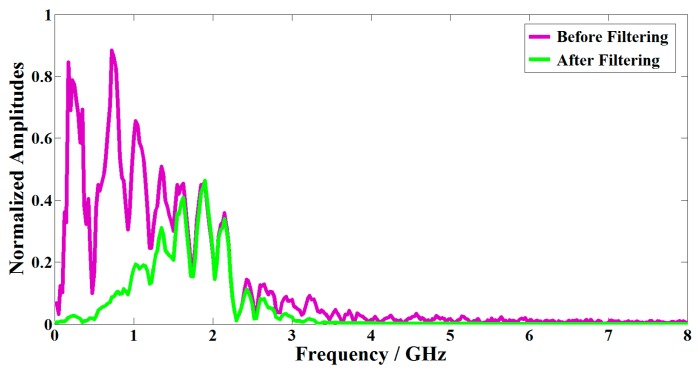
The frequency spectrum before (**purple**) and after (**green**) the band-pass filtering.

A band-pass filter in line with the characteristics of GPR data could be an effective tool to improve the signal-to-noise ratio (SNR). For example, the designed central frequency and bandwidth of the two G air-coupled GPRs were both 2 GHz, respectively. Therefore, we chose a band-pass filter with a pass band of 1–3 GHz (Figure 3).

#### 2.2.4. Amplitude Compensation

The GPR antenna can be approximated to an oscillating dipole that radiates spherical waves. The amplitude of the electromagnetic wave that propagates in the ground suffers, therefore, a geometric attenuation due to the distribution of the energy on the front of spherical wave.

The amplitude A(r) at the distance *r* is given as follows:

(2) $$A(r)=G(r)A_{0}e^{-\alpha r}$$

where $A_{0}$ is the value of the initial amplitude transmitted from GPR antenna, *α* is the attenuation constant, G(r) is the factor of geometrical spreading which can be derived by Equation (3):

(3) $$G(r)=\frac{1}{r}$$

Therefore, the amplitude can be compensated by means of multiplying the GPR signals by 1/G(r).

#### 2.2.5. Horizontal High Pass Filtering

**Figure 4:**
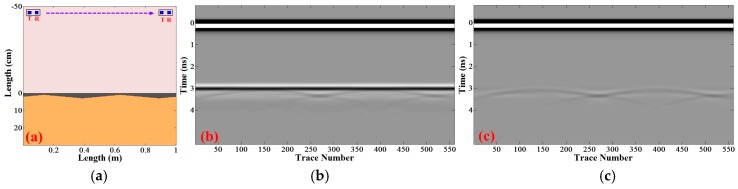
(**a**) The model for the horizontal high-pass filtering; (**b**) the simulated GPR signals; (**c**) the processed GPR signals after horizontal high-pass filtering.

Generally, the designed thickness for the top surface layer of highways in China is 3–5 cm, while the resolutions for 1 GHz and 2 GHz GPR in asphalt layers are 6–8 cm and 3–4 cm, respectively, which makes it difficult to separate the echoes from the top and bottom surface of the top surface layer directly. Thus, an effective method is necessary for extracting the top surface layer from the GPR data. The top surface layer seems to be not completely smooth due to the limitations of pavement construction technology, which makes it possible for us to distinguish the top surface layer. The reflections from the top surface of the top surface layer were leveled first, and then the trailing of the top surface reflections became the low frequency components and the bottom surface reflections became the high frequency components in the horizontal direction. We employed a high pass filter to process the GPR data in the horizontal direction and the bottom surface reflections would be extracted from the top surface reflections. A simple model was simulated to test the effect of the method as shown in Figure 4. It could be seen that the obscured thin layer became clear.

### 2.3. Layer Localization Method

**Figure 5:**
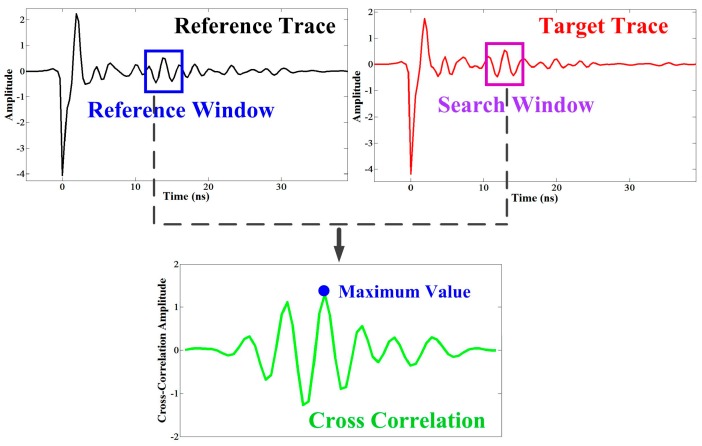
The schematic for step (2) of the modified trace correlation method. The black signal and red signal indicate reference trace and target trace respectively. The blue and purple rectangles indicate reference window and search window respectively. The search point is the center of both reference window and search window. The green line indicates the cross correlation between signals within reference window and search window. The blue point is maximum value of correlation.

Because of the high pulse repetition frequency (PRF) of GPR, the GPR echoes of two adjacent traces from the same interface have similar time delay and amplitude, in other words, good correlation. Thus, we can take advantage of the good correlation of two adjacent traces to localize the underground media interface. However, buried objects or other underground interferences would influence the localization effect. In order to suppress interference and extract subsurface layers accurately, we put forward a modified trace correlation method which includes the following steps:

(1) Determination of initial values
  Regard the first trace as reference trace and determine appropriate values for length of the reference window and search window, correlation threshold ($T_{C}$) and time threshold ($T_{L}$), and then determine the initial search point ($P_{1}$) by visual inspection.
(2) Calculation of correlation
  Take the next trace as target trace and calculate the correlation between the signal in the reference window of the reference trace and the signal in the search window of the target trace, and then determine the maximum value of the correlation ($M_{C}$) and the corresponding point ($P_{2}$) (Figure 5).
(3) Determination of layer position
  If $M_{C}>T_{C}$ and $\left|P_{2}-P_{1}\right|<T_{L}$, then consider $P_{2}$ as the layer position of the target trace, also as the new search point, regard the target trace as a new reference trace. If $M_{C}<T_{C}$, or $M_{C}>T_{C}$ but $\left|P_{2}-P_{1}\right|>T_{L}$, then consider the nearest extreme point from $P_{1}$ as the layer position of target trace, but keep the preceding reference trace and search point.
(4) Implement steps (2) and (3) to all the traces in order.

### 2.4. Estimation of Pavement Thickness

**Figure 6:**
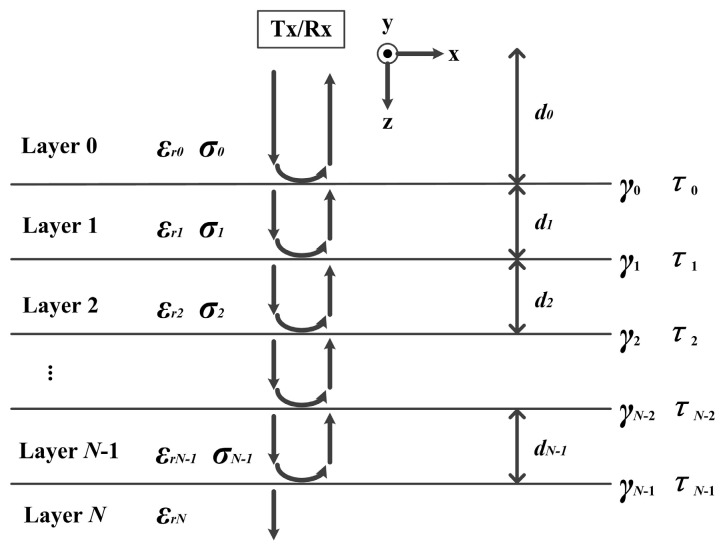
The planar layer model of asphalt pavements. $\varepsilon_{r,i}$, $\sigma_{i}$, $d_{i}$ are, respectively, the permittivity, conductivity and thickness at layer *i*, $\gamma_{i}$ and $\tau_{i}$ are reflection and transmission coefficient at layer interface *i*.

The asphalt pavement system could be modeled as a set of N + 1 homogeneous planar layers, as shown in Figure 6. Each layer *i* was assumed to be a semi-infinite layer that has a finite thickness $d_{i}$, a dielectric constant $\varepsilon_{r,i}$, a conductivity $\sigma_{i}$, and a magnetic permeability equal to that of free space because of the nonmagnetic nature of pavement materials [30,31].

The reflection coefficients ($\gamma_{i}$) and transmission coefficients ($\tau_{i}$) at each layer interface *i* could be described as follows [32]:

(4) $$\gamma_{i}=\frac{\sqrt{\varepsilon_{r,i}}-\sqrt{\varepsilon_{r,i+1}}}{\sqrt{\varepsilon_{r,i}}+\sqrt{\varepsilon_{r,i+1}}}$$

(5) $$\tau_{i}=\frac{2\sqrt{\varepsilon_{r,i}}}{\sqrt{\varepsilon_{r,i}}+\sqrt{\varepsilon_{r,i+1}}}=1+\gamma_{i}$$

Substituting the reflection and transmission coefficients in the model yields the reflection amplitudes $A_{n}$ at interface *n* as Equation (6):

(6) $$A_{n}=A_{m}\cdot \frac{\sqrt{\varepsilon_{r,n}}-\sqrt{\varepsilon_{r,n+1}}}{\sqrt{\varepsilon_{r,n}}+\sqrt{\varepsilon_{r,n+1}}}\left[\prod_{i=0}^{n-1}\left(1-\gamma_{i}^{2}\right)\right]$$

where $A_{m}$ is amplitude of the incident signal.

Thus, the end result for dielectric constant of *n*th layer ($\varepsilon_{r,n}$) could be found by transforming Equation (6), as given by the next equation:

(7) $$\varepsilon_{r,n}=\varepsilon_{r,n-1}{\left(\frac{1-{\left(\frac{A_{0}}{A_{m}}\right)}^{2}-\sum_{i=1}^{n-2}\gamma_{i}\frac{A_{i}}{A_{m}}-\frac{A_{n-1}}{A_{m}}}{1-{\left(\frac{A_{0}}{A_{m}}\right)}^{2}-\sum_{i=1}^{n-2}\gamma_{i}\frac{A_{i}}{A_{m}}+\frac{A_{n-1}}{A_{m}}}\right)}^{2}\;,\;for\;n=2,\cdots ,N$$

The dielectric constant of the surface layer could be derived briefly as follows:

(8) $$\varepsilon_{r,1}={\left(\frac{1+A_{0}/A_{m}}{1-A_{0}/A_{m}}\right)}^{2}$$

In order to obtain the values of the dielectric constant, we needed both the amplitudes of the surface reflected pulses and the amplitude of the GPR incident signal. However, only the amplitudes of the surface reflected pulses could be ascertained. Therefore, we designed a calibration experiment to get the incident signal. The GPR was placed on a large metal plate and switched to work. In this case, the amplitude of reflected signal could be considered as the amplitude of the incident signal, and then the dielectric constant could be obtained [33]. According to the calculated dielectric constant, the velocity of the electromagnetic wave propagation through the *n*th layer of pavement was determined as:

(9) vn=cεr,n

where, *c* is the speed of light in vacuum, $\varepsilon_{r,n}$ is the dielectric constant of *n*th layer.

Finally, the accurate thicknesses of asphalt pavement layers (d) could be determined from the dielectric constant ($\varepsilon_{r}$) and delay time of the latter echo relative to the former echo (t) [34]:

(10) $$d=\frac{vt}{2}=\frac{ct}{2\sqrt{\varepsilon_{r}}}$$

### 2.5. Compaction Degree Detection Method

There is strong relationship between compactness and density, the density also has great impact on the dielectric constant of asphalt pavement. Thus, it is feasible to detect the uncompaction of asphalt pavement using the relationship between the dielectric constant and compactness. Previous studies made testing asphalt models to get the fitting relationship between the dielectric constant and compactness [35,36], but the fitting relationship were not suitable for practical application due to the differences in dielectric constant between lab models and pavements or between different pavements. Therefore, we introduced a compaction factor in order to overcome the influence of those differences by considering the general value of a certain pavement. In this condition, although the dielectric constants between lab models and pavements or between two pavements were different, the poorly compacted area would show a smaller dielectric constant than the average. The compaction factor was defined as Equation (11):

(11) Ic=εrεr¯

where, for pavements, $\varepsilon_{r}$ is the dielectric constant of a certain location, $\bar{\varepsilon_{r}}$ is the average dielectric constant of the pavement; while for lab sample, $\varepsilon_{r}$ and $\bar{\varepsilon_{r}}$ are the dielectric constant of a certain lab asphalt sample and standard lab sample, respectively.

**Figure 7:**
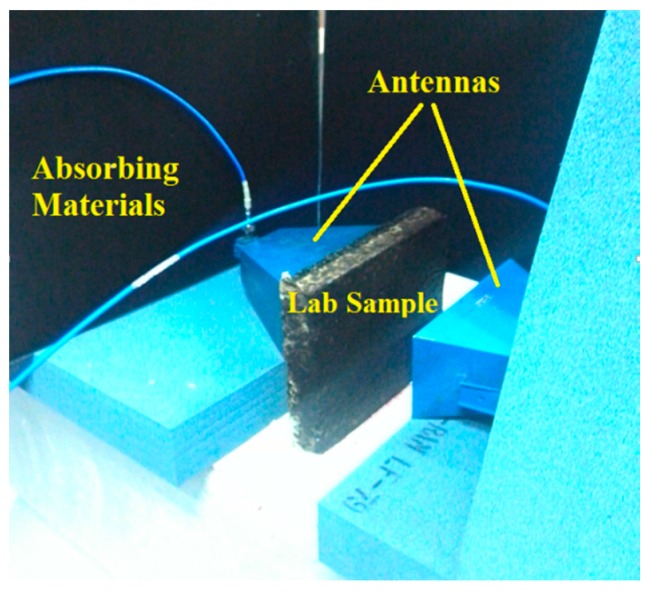
The testing system for the dielectric constant of lab samples.

We made seven test asphalt samples with different compaction degrees and densities, and then the dielectric constant of each lab sample could be measured using the system as shown in Figure 7 on the basis of the propagation velocity of electromagnetic wave. The system was composed of a vector network analyzer, transmit and receive antennas, and a coaxial cable. Absorbing materials were used for reducing the environmental noise. Each lab sample was measured ten times and the density, compaction degree, and the measured dielectric constant with 3σ error were obtained as shown in Table 1.

**Figure 8:**
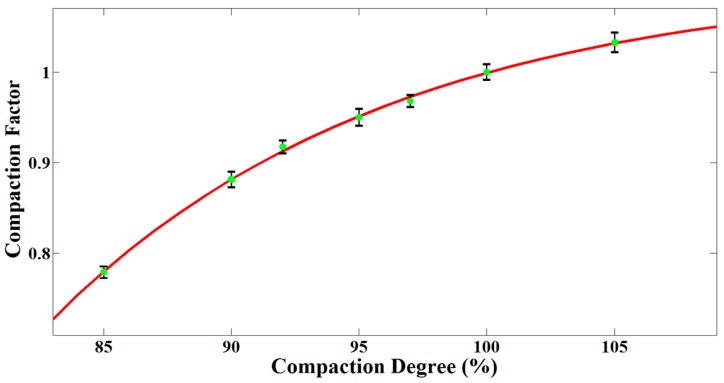
The fitting relationship between compaction degree and the compaction factor. The green dots are compaction factors calculated from measured dielectric constants. The error bars show the triple standard derivation. The red curve indicates the fitting function.

According to the compaction degree and the measured dielectric constant, we used exponential curve fitting to determine the relationship between compaction degree and the compaction factor in Figure 8. The fitting function was given as follows:

(12) $$I_{compact}=0.397+0.343e^{1.396I_{c}}+2.725e^{-1.396I_{c}}$$

where, $I_{compact}$ indicates the compaction degree, $I_{c}$ is the compaction factor.

Based on the fitting relationship, a lower limit of the qualified compaction degree in practice determines a corresponding compaction factor threshold. We can compare the calculated compaction factor of the pavement with the threshold, and then retest the unqualified region. In addition, it is worth noting that the method is mainly used for detecting the uncompaction because we defined the compaction factor with the mean dielectric constant of the pavement. As a result, some values of compaction factor greater than 1 do not indicate overcompaction of the pavement.

### 2.6. Delamination Detection Method

**Figure 9:**
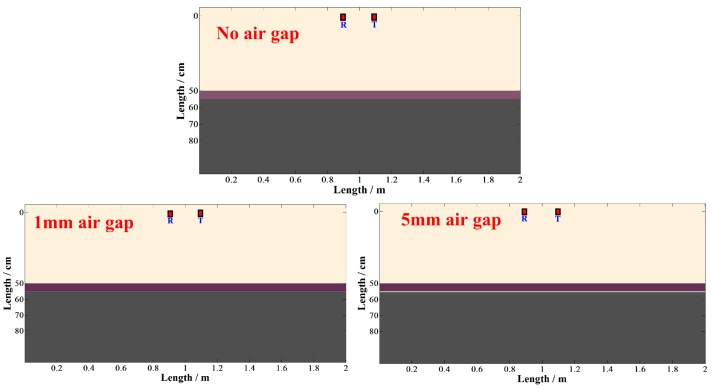
The models of delamination at interface. The given thicknesses of air gaps are 0 mm, 1 mm and 5 mm, respectively.

Generally, the delamination thickness is less than 2 cm, which differs from an underground cavity, so GPR cannot obtain the echo of such a thin air gap because of the resolution limitations. However, it is inevitable that the reflection signal of pavement with air gaps is different from that of standard pavement. Thus, we attempted to analyze the distinction caused by the thin air gap to detect the delamination. A two planar layer model was developed to simulate the GPR signal, the thickness of the upper layer is 4 cm and the dielectric constants of the two layers are 6 and 7, respectively. The distance from the antennas to ground and the distance between the transmit and receive antennas are 50 cm and 20 cm, respectively. We simulated three conditions by adjusting the distance between two layers, i.e., the thickness of air gap, including 0, 1 mm and 5 mm (Figure 9), and then the simulated GPR signals could be obtained.

**Figure 10:**
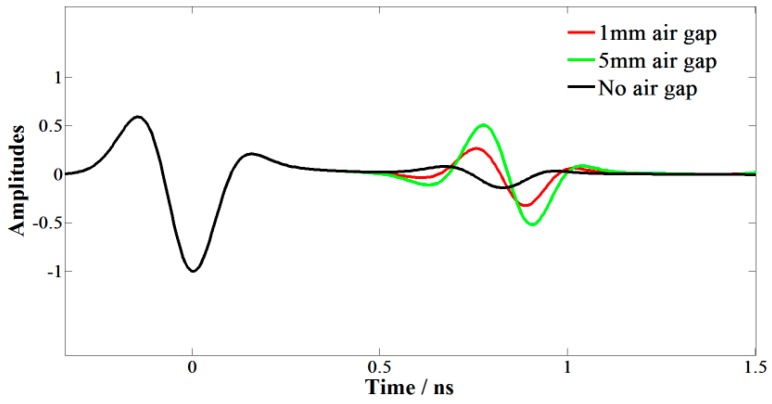
The simulated GPR signals of the three models in Figure 9.

As shown in Figure 10, the top and bottom surface of the air gap could not be separated, but the amplitudes of reflection signals obviously increased compared with those of the standard model. To identify the distinction of GPR signals caused by delamination, we developed a delamination factor which was defined as:

(13) $$I_{a}=\frac{A_{1}}{A_{0}}$$

where, $I_{a}$ indicates the delamination factor, $A_{0}$ and $A_{1}$ are amplitudes of echoes from the top layer and the bottom layer, respectively.

According to the Fresnel reflection/refraction model and electromagnetic wave propagation theory, $I_{a}$ could be expressed by the dielectric constants of the top layer ($\varepsilon_{1}$) and the bottom layer ($\varepsilon_{2}$):

(14) $$I_{a}=\frac{A_{1}}{A_{0}}=\frac{\sqrt{\varepsilon_{2}}-\sqrt{\varepsilon_{1}}}{\sqrt{\varepsilon_{2}}+\sqrt{\varepsilon_{1}}}\left(\frac{\sqrt{\varepsilon_{1}}+1}{\sqrt{\varepsilon_{1}}-1}-\frac{\sqrt{\varepsilon_{1}}-1}{\sqrt{\varepsilon_{1}}+1}\right)$$

Generally, the dielectric constants of two adjacent asphalt layers were similar and the relationship $\varepsilon_{2}<2\varepsilon_{1}$ was satisfied [37]. In this condition, $I_{a}$ could be derived as follows:

(15) $$I_{a}<\frac{0.686\sqrt{\varepsilon_{1}}}{\varepsilon_{1}-1}$$

Furthermore, if the air gap is thick enough, i.e., an underground cavity, $I_{a}$ could be given by substituting $\varepsilon_{2}=1$ into Equation (14) as following equation:

(16) $$I_{a}=1-{\left(\frac{\sqrt{\varepsilon_{1}}-1}{\sqrt{\varepsilon_{1}}+1}\right)}^{2}$$

Therefore, we could obtain a warning value range of the delamination factor possibly caused by air gaps according to Equations (15) and (16):

(17) $$\frac{0.686\sqrt{\varepsilon_{1}}}{\varepsilon_{1}-1}\leq I_{a}\leq 1-{\left(\frac{\sqrt{\varepsilon_{1}}-1}{\sqrt{\varepsilon_{1}}+1}\right)}^{2}$$

In field tests, the delamination factor and the warning value range could be calculated from the GPR data, and then the region whose delamination factor was within the warning value range should be retested by other methods such as core-drilling.

## 3. Results

**Figure 11:**
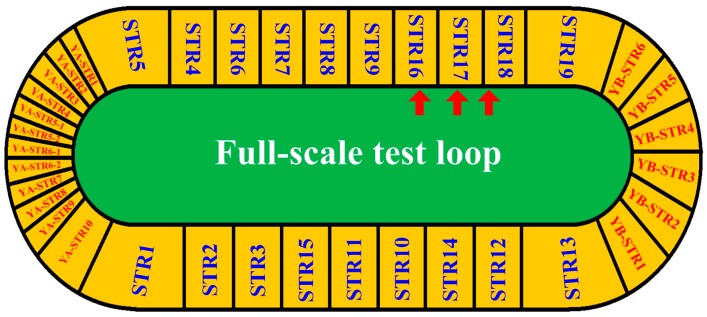
The full-scale test loop in the highway proving ground of Ministry of Transport of China. The red arrows indicate the drilling positions of field cores.

Based on the vehicle-mounted GPR detection system, we could estimate the thicknesses of top surface layer and surface layer of asphalt pavements, and also detected some possible defects (i.e., uncompaction and delamination) using the signal processing methods and the defects detection methods we presented. In order to test the performance of the detection system and methods, we carried out a field test on a full-scale test loop in the highway proving ground of Ministry of Transport of China. The total length and width of the test loop are 2039 m and 12 m, respectively (Figure 11). STR and YB-STR are asphalt concrete pavements that conclude 25 structures, while YA-STR is reinforced concrete pavements. The full-scale test loop was completed in 2016, which corresponds to newly-constructed pavement. Therefore, it is more difficult to detect the pavement structure by GPR due to the tight bond between each two asphalt layers. Besides, the pavement structures of the test loop are various and more intricate than the majority of pavements in use, so the field test is enough to prove the suitability and accuracy of our system and methods.

**Figure 12:**
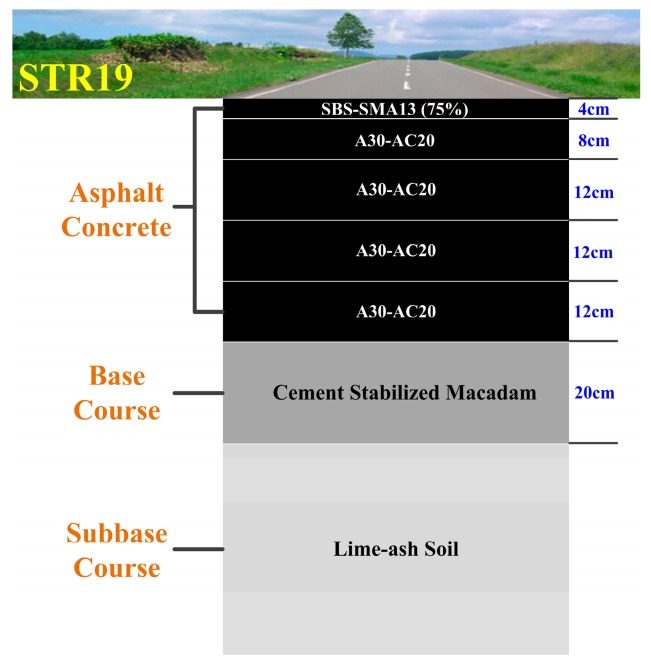
The cross-section of STR19.

We collected ~ten GPR signal traces per meter on the full-scale test loop and processed the data of each pavement segment. We take here the most complicated structure (STR19) as an example. Its cross section is shown in Figure 12.

**Figure 13:**
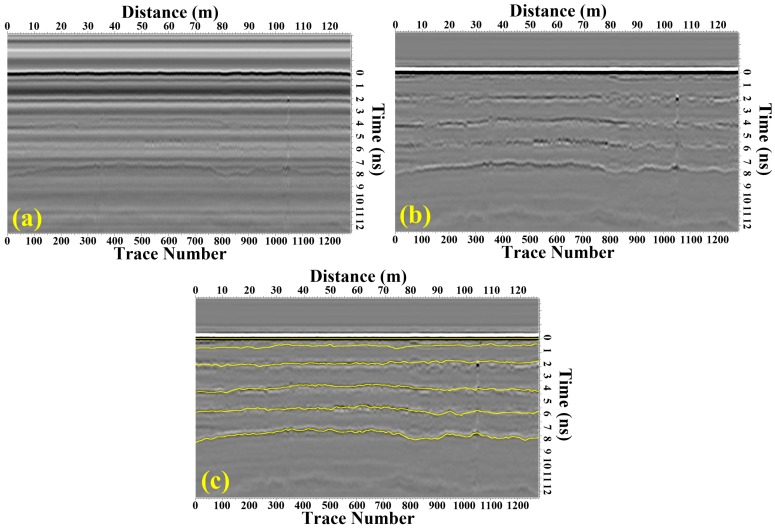
(**a**) The raw GPR data of STR 19; (**b**) The processed GPR data; (**c**) The underground layers of STR19.

**Figure 14:**
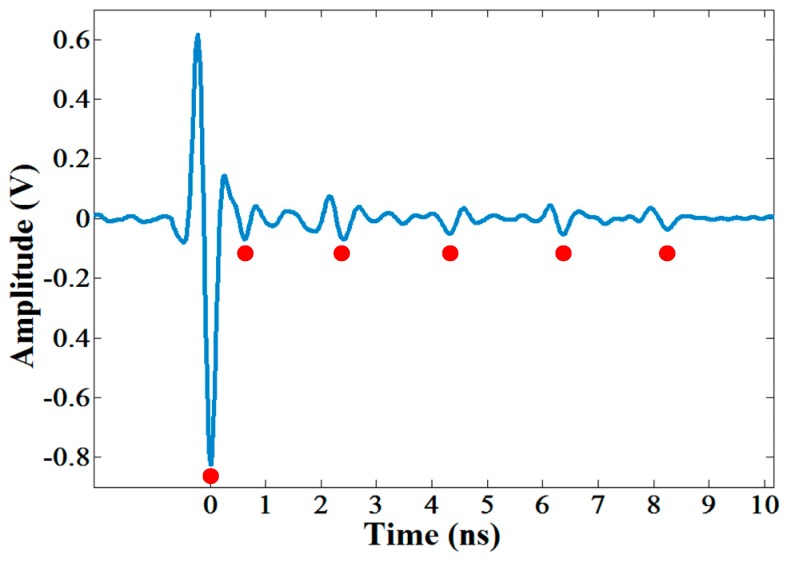
A single trace of the processed GPR data of STR19. The red dots indicate the locations of echoes from six interfaces.

The surface asphalt layer of STR19 was composed of five sub-layers and the thicknesses of top surface layer and surface layer were 4 cm and 48 cm, respectively. The raw GPR image was shown in Figure 13a, we processed the data using the methods introduced in Section 2.2 and obtained the processed GPR image with improved signal to noise ratio (SNR) and resolution (Figure 13b). Five sub-layers of the surface asphalt layer could be obviously distinguished in the processed GPR image, and the echoes from ground and each interface between two layers could also be distinguished in a single trace (Figure 14). Furthermore, we applied the modified trace correlation method introduced in Section 2.3 to localize each layer, and then the time delays and amplitudes of each reflection layer could be obtained (Figure 13c). With the algorithm illustrated in Section 2.4, the thicknesses of the top surface layer and surface layer were estimated to be 3.88 cm and 46.82 cm with a standard derivation of 0.21 cm and 2.46 cm, respectively, which are in good agreement with the designed thicknesses of STR19.

**Figure 15:**
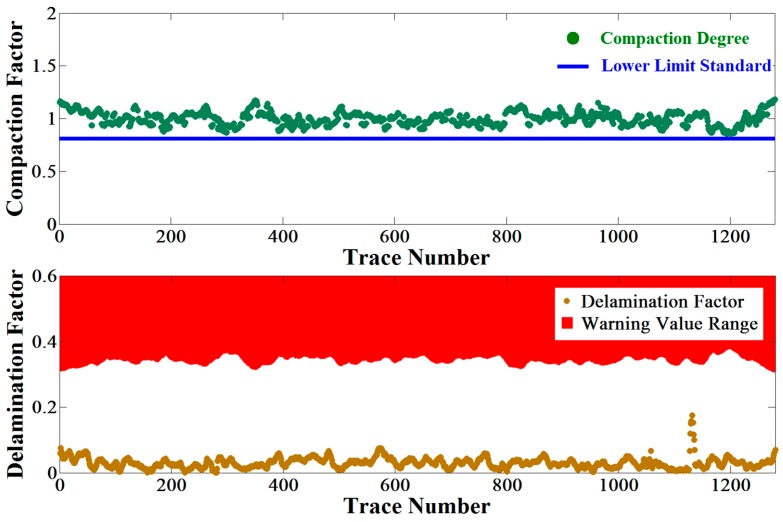
The compaction factors and delamination factors of STR19.

In addition, we calculated the two factors of STR19 trace by trace as shown in Figure 15 according to the definitions of compaction factor and delamination factor presented in Section 2.5 and Section 2.6. Hypothetically, the compaction degree of asphalt pavement was required to be no less than 85%, which constrained the lower limit of the compaction factor to be 0.78 based on Equation (12). It could be seen that all the compaction factors are greater than the lower limit, and also all the delamination factors are beyond the calculated warning value range by Equation (13). The results indicated that STR19 is probably a section of qualified asphalt pavement without uncompaction and air gap.

In the same way, we analyzed the GPR data of each segment of the full-scale test loop and estimated the thicknesses of top surface layer and surface layer. The mean estimated result, the standard deviation, and the relative error to the designed thickness of each subsection are listed in Table 2.

**Figure 16:**
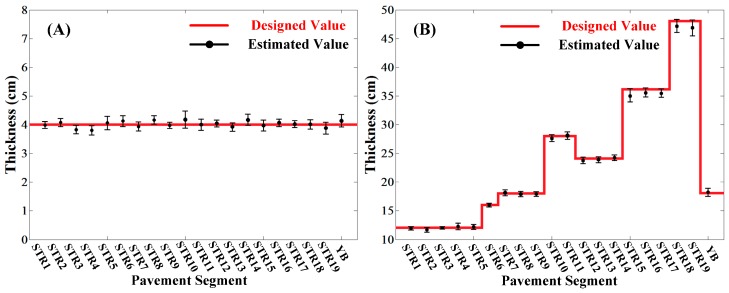
The comparison of designed thicknesses and estimated thicknesses of each pavement segments. (**A**) The top surface layer; (**B**) The surface layer.

**Figure 17:**
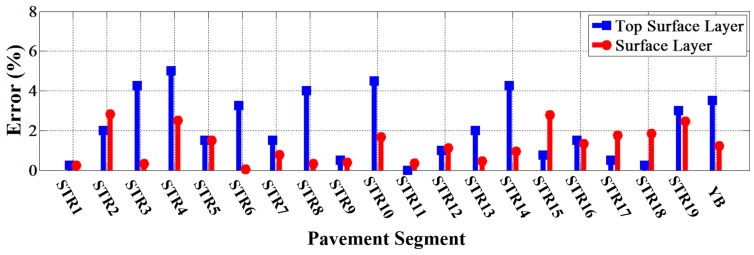
The estimated errors of each pavement segments.

The relationships between designed thickness and estimated thickness of both the top surface layer and surface layer of each pavement segment were analyzed and are shown in Figure 16. It could be seen that all the estimated results were in good agreement with the designed thicknesses. Furthermore, as shown in Figure 17, the relative errors to the designed thicknesses of the top surface layer and surface layer of all the segments were less than 5% and 3%, respectively, which showed a satisfactory accuracy in continuous detection.

**Figure 18:**
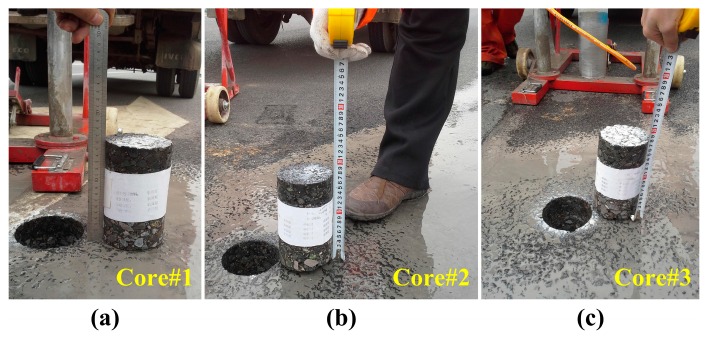
The field cores of asphalt surface layer from the test loop in (**a**) STR7; (**b**) STR8; (**c**) STR9.

Furthermore, as marked in Figure 11, three field cores of asphalt surface layer were drilled from the test loop in STR6, STR7 and STR8, respectively (Figure 18).

**Figure 19:**
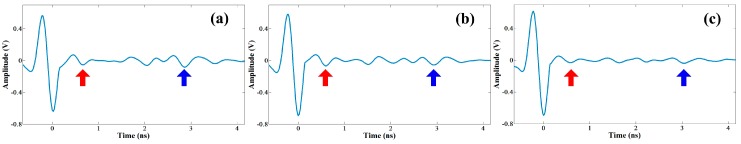
The corresponding GPR signal for (**a**) core#1; (**b**) core#2; (**c**) core#3. The red and blue arrows indicate the reflections from top surface layers and surface layers, respectively.

According to the localization, three corresponding GPR signals were extracted as shown in Figure 19, and then the estimated thicknesses of the surface and top surface layer could be obtained. Table 3 shows the comparison between estimated results from GPR and measured results from filed cores. It could be seen that the estimated errors of both top surface and surface layer were less than 5%, which also proved a satisfactory accuracy in local detection.

## 4. Discussion

The vehicle-mounted GPR detection system we developed in this paper integrated the air-coupled GPR, ground-coupled GPR, GPS and cameras. Compared with the common vehicle-mounted GPR system [7,8,9,10], it satisfied more comprehensive requirements of pavement detection with three advantages: (1) the detection range could cover both deep and shallow pavement structures by using a wide bandwidth from 300 MHz to 2 GHz; (2) the locations of collected data could be determined with the GPS, which was beneficial to seek and recheck the possible defects; (3) the corresponding surface photos from cameras contributed to the analysis of GPR data and the judgments of possible road defects. The structure and stability of the vehicle-mounted GPR system had been verified by many field tests and the system could be applied for the asphalt pavement detection, even for the highway detection.

The test loop we used for the field test was a new-constructed pavement with high construction standards and ~22,000 traces of GPR data for the test loop were collected during the field test. Different from the analysis of one trace in previous studies [15,16,17], the thin top surface layer of the whole pavement was extracted and the thicknesses of the top surface layer and surface layer were estimated. On one hand, the predicted thicknesses of surface layer and top surface layer for each segment were consistent with the designed values, which showed an acceptable effect in continuous detection. On the other hand, the comparison between measured results from field cores and predicted results from GPR suggested a satisfactory accuracy in local detection. The results of field test proved that the predicted errors for the surface layer and top surface layer are less than 3% and 5%, respectively, which could satisfy the accuracy requirements of asphalt pavement detection.

The compaction degree was related to the density and air voids which would influence the dielectric constant of the asphalt pavement. Previous studies attempted to determine the relationships between the dielectric constant and density or air voids by lab experiments [18,19,20,21,22,23,24]. However, fitting the relationship between compaction degree and dielectric constant using lab samples directly might be not appropriate for the following reasons: (1) the differences between lab samples and asphalt pavements; (2) the differences between various pavements; (3) the differences for a certain pavement under different weather conditions. Therefore, the compaction factor could reduce such impacts by allowing for the general level of the whole pavement. The poorly compacted area would lead to a smaller compaction factor no matter how the condition of a pavement was. Actually, the method was not developed to estimate the exact degree of compaction of pavements, but rather to detect the possible poorly compacted areas in asphalt pavements.

However, it should be noted that the detection methods for the inadequate compaction and delamination were based on model simulation and the principles of electromagnetic wave propagation. The actual pavement defects could contribute to the determination of the warning value range, so the methods required quantities of real road defects and matched GPR data to optimize the parameters and improve the detection effects. The more field cores with defects that are drilled, the higher the precision of the presented methods would be. The GPR results and field cores from the test loop did not indicate any possible inadequate compaction or delamination. Therefore, future studies will focus on the applications and drillings in used asphalt pavements.

## 5. Conclusions

In this paper, we concentrated on the thickness estimation and defects detection of asphalt pavements. The main contents could be concluded that:

(1) The proposed vehicle-mounted GPR detection system was applicable for the asphalt pavements. The system could not only detect the thicknesses and defects of pavement by air-coupled and ground-coupled GPRs, but also provide the accurate location and surface photos by GPS and cameras.
(2) Continuous thin top surface layer could be extracted from GPR data which had an inadequate resolution using a horizontal high-pass filter to GPR data and a modified trace correlation method.
(3) A compaction factor and a delamination factor were developed and used for detecting the possible poorly compacted and delaminated areas of asphalt pavement.
(4) In field tests, the predicted thicknesses suggested a satisfactory accuracy according to both continuous estimated results and local estimated results. The predicted errors of the surface layer and top surface layer were less than 3% and 5%, respectively, which could satisfy the requirements of asphalt pavement detection. The results indicated that the system and methods proposed in this paper show satisfactory performance in asphalt pavement detection.

## Figures and Tables

**Table 1:** The parameters and measured dielectric constants of lab samples.

Size (mm)	Number	Compaction Degree (%)	Density (g/cm^3^)	Dielectric Constant
**300 × 300 × 50**	1	85	2.070	5.102 ± 0.041
	2	90	2.192	5.770 ± 0.049
	3	92	2.240	6.007 ± 0.041
	4	95	2.313	6.221 ± 0.050
	5	97	2.362	6.336 ± 0.038
	6	100	2.435	6.546 ± 0.050
	7	105	2.557	6.761 ± 0.067

**Table 2:** The estimated thicknesses and relative errors of the top surface layer and surface layer of each pavement segment.

Pavement Segments	Top Surface Layer				Surface Layer			
	Designed Thickness (cm)	Estimated Thickness (cm)	Standard Deviation (cm)	Relative Error (%)	Designed Thickness (cm)	Estimated Thickness (cm)	Standard Deviation (cm)	Relative Error (%)
STR1	4.00	3.99	0.12	0.25	12.00	11.97	0.30	0.25
STR2	4.00	4.08	0.14	2.00	12.00	11.66	0.40	2.83
STR3	4.00	3.83	0.14	4.25	12.00	12.04	0.18	0.33
STR4	4.00	3.80	0.16	5.00	12.00	12.30	0.57	2.50
STR5	4.00	4.06	0.23	1.50	12.00	12.18	0.43	1.50
STR6	4.00	4.13	0.19	3.25	16.00	16.01	0.32	0.06
STR7	4.00	3.94	0.16	4.00	18.00	18.14	0.51	0.78
STR8	4.00	4.16	0.15	1.50	18.00	17.94	0.46	0.33
STR9	4.00	3.98	0.11	0.50	18.00	17.93	0.41	0.39
STR10	4.00	4.18	0.30	4.50	28.00	27.53	0.44	1.68
STR11	4.00	4.00	0.19	0.00	28.00	28.10	0.64	0.36
STR12	4.00	4.04	0.12	1.00	24.00	23.73	0.53	1.12
STR13	4.00	3.92	0.15	2.00	24.00	23.89	0.51	0.46
STR14	4.00	4.17	0.20	4.25	24.00	24.23	0.48	0.96
STR15	4.00	3.97	0.19	0.75	36.00	35.00	1.03	2.78
STR16	4.00	4.06	0.13	1.50	36.00	35.52	0.68	1.33
STR17	4.00	4.02	0.12	0.50	36.00	35.37	0.60	1.75
STR18	4.00	4.01	0.16	0.25	48.00	47.11	1.07	1.85
STR19	4.00	3.88	0.21	3.00	48.00	46.82	1.33	2.46
YB-STR	4.00	4.14	0.22	3.00	18.00	18.22	0.72	1.22

**Table 3:** The comparison between estimated results from GPR and measured results from field cores.

Cores	Top Surface Layer			Surface Layer			Poorly Compacted		Delamination	
	Measured Thickness (cm)	Estimated Thickness (cm)	Error (%)	Measured Thickness (cm)	Estimated Thickness (cm)	Error (%)	Estimated Result	Measured Result	Estimated Result	Measured Result
#1	4.0	4.1	2.5	16.2	16.6	2.5	×	×	×	×
#2	3.9	3.8	2.5	17.8	17.6	1.1	×	×	×	×
#3	3.9	3.7	5.0	18.3	17.8	2.7	×	×	×	×
